# The performance of the progressive resolution optimizer (PRO) for RapidArc planning in targets with low‐density media

**DOI:** 10.1120/jacmp.v14i6.4382

**Published:** 2013-11-04

**Authors:** Monica W.K. Kan, Lucullus H.T. Leung, Peter K.N. Yu

**Affiliations:** ^1^ Department of Oncology Princess Margaret Hospital Hong Kong SAR; ^2^ Department of Physics and Materials Science City University of Hong Kong Kowloon Tong Hong Kong SAR

**Keywords:** RapidArc, optimization algorithm, Acuros XB algorithm, low‐density media, air cavity correction

## Abstract

A new version of progressive resolution optimizer (PRO) with an option of air cavity correction has been implemented for RapidArc volumetric‐modulated arc therapy (RA). The purpose of this study was to compare the performance of this new PRO with the use of air cavity correction option (PRO10_air) against the one without the use of the air cavity correction option (PRO10_no‐air) for RapidArc planning in targets with low‐density media of different sizes and complexities. The performance of PRO10_no‐air and PRO10_air was initially compared using single‐arc plans created for four different simple heterogeneous phantoms with virtual targets and organs at risk. Multiple‐arc planning of 12 real patients having nasopharyngeal carcinomas (NPC) and ten patients having non‐small cell lung cancer (NSCLC) were then performed using the above two options for further comparison. Dose calculations were performed using both the Acuros XB (AXB) algorithm with the dose to medium option and the analytical anisotropic algorithm (AAA). The effect of using intermediate dose option after the first optimization cycle in PRO10_air and PRO10_no‐air was also investigated and compared. Plans were evaluated and compared using target dose coverage, critical organ sparing, conformity index, and dose homogeneity index. For NSCLC cases or cases for which large volumes of low‐density media were present in or adjacent to the target volume, the use of the air cavity correction option in PROIO was shown to be beneficial. For NPC cases or cases for which small volumes of both low‐ and high‐density media existed in the target volume, the use of air cavity correction in PRO10 did not improve the plan quality. Based on the AXB dose calculation results, the use of PRO10_air could produce up to 18% less coverage to the bony structures of the planning target volumes for NPC cases. When the intermediate dose option in PRO10 was used, there was negligible difference observed in plan quality between optimizations with and without using the air cavity correction option.

PACS number: 87.55.D‐, 87.55.de, 87.56.N‐

## I. INTRODUCTION

Recently, RapidArc (Varian Medical Systems, Palo Alto, CA) has been found to produce comparable conformal dose distributions and faster treatment delivery compared to conventional static intensity‐modulated radiotherapy (IMRT) in various treatment sites, some of which involved low‐density medium such as lung, breast, and nasopharyngeal regions.^1^, ^2^, ^3^, ^4^, ^5^

Our previous study also reported better treatment efficiency and plan quality achieved using RapidArc (RA) compared to IMRT for early stage nasopharyngeal carcinoma (NPC).^(2)^ The study of Verbakel et al.^(4)^ showed that RA allowed much faster delivery of hypofractionated doses and generated more conformal plans than conventional stereotactic body radiotherapy (SBRT) for peripheral lung tumors.

The progressive resolution optimizer (PRO) of RA allows variation of the multileaf collimator's (MLC) leaf positions, gantry rotation speed, and dose rate to produce the optimal dose distribution for inverse planning. In order to provide fast dose calculation during the optimization process, a simplified multiresolution pencil beam photon dose calculation algorithm (MRDC) is used. The MRDC is less accurate than the final dose calculation algorithms such as the Acuros XB algorithm (AXB) and the analytical anisotropic algorithm (AAA) when accounting for the effects of tissue heterogeneities. As a result, discrepancy will be observed between the target doses shown in the optimizer and that of final dose calculations. The study by Ong et al.^(5)^ reported a lower average planning target volume (PTV) dose calculated by AAA for lung tumors when compared to that calculated by the MRDC at the end of the optimization. Their study used an early version of PRO (version 8.2). They used a PTV dose of 5% to 10% higher than the prescribed dose when setting optimization objectives to compensate for dose differences between the MRDC and AAA. In order to minimize such discrepancy, the newly released PRO (version 10.0) allowed the use of an air cavity correction option that applies a finer resolution in the internal dose calculation grid during optimization when air equivalent densities are identified. Vanetti et al.^(6)^ compared the performance of the earlier and more recent versions of PRO algorithm and reported that, with the use of air cavity correction option together with the use of intermediate dose option in the second run of optimization, the dose‐volume histogram (DVH) shown in optimization became almost identical to that of the AXB calculation in lung cases. However, the effect of using the air cavity correction option and the intermediate dose option separately was not investigated. The effect of using the air cavity correction option alone on targets with different sizes and complexity levels of heterogeneity was not previously reported. Our current study focused on more detailed and extensive comparison between PRO of version 10.0 (PRO10) with and without the use of the air cavity correction option, in RA planning for targets with low‐density heterogeneities, including the presence of air and lung. The plan quality from single‐arc plans of simple geometric virtual phantoms with air cavities of different sizes and complexities to multiple‐arc plans of real patients having NPC and non‐small cell lung cancer (NSCLC) was evaluated. The effect of different optimization options on target coverage was studied by stratifying the targets into components of different densities. For the study of NSCLC, the target was divided into components in tissue and in lung for evaluation. For the study of NPC, the target was divided into components in tissue, air, and bone for evaluation. The effect of using the air cavity correction option in NPC was selected in the current study mainly because of the presence of small and complex shape of air cavities and bony structures. Furthermore, the effect of using the intermediate dose options in the second run of optimization after the first run of initial optimization with and without turning on the air cavity correction option on plan quality was investigated.

## II. MATERIALS AND METHODS

All RA plans were generated using 6 MV photon beams and modulated with 120 multileaf collimators from a linear accelerator (Clinac 23EX; Varian Medical Systems). Optimizations and dose calculations were performed with the Eclipse treatment planning system (version 10.0). All plans were initially generated and optimized based on a deterministic dose calculation algorithm, AXB, using the dose‐to‐medium option with 2.5 mm grid resolution. It was selected for the current study because several previous studies proved that AXB was able to produce dose calculation accuracy comparable to that of the Monte Carlo simulations in heterogeneous media, especially in the presence of low‐density media.^7^, ^8^, ^9^, ^10^, ^11^, ^12^ Furthermore, these generated plans were recalculated using AAA with 2.5 mm grid resolution to investigate if there would be any difference in the comparison result due to the conversion of dose calculation to a commonly used model‐based algorithm, which produced less accurate dose calculation compared with AXB in the presence of heterogeneous medium.

### A. Optimization algorithm

The progressive resolution optimizer implemented in our existing Eclipse treatment planning system included an earlier version of 8.6.15 (PRO8) and a more recent version of 10.0.28 (PRO10) (Eclipse, Varian Medical Systems). Both versions were implemented based on a publication by Otto.^(13)^ For PRO8, the optimization process proceeds through five multiresolution levels, in which both the number of control points and dose calculation sectors are increased at each level, progressively, from 10 to 177. For PRO10, the optimization process proceeds through four multiresolution levels. All control points are optimized at the same time during all levels, while only the resolution of dose calculation sectors are refined progressively from the first to fourth level. Since a lower density of control points is included in PRO8 optimization during the early levels of optimization, the optimal modulated pattern might not be properly modeled. Unlike PRO8, PRO10 estimates the achievable dose distribution during the earlier levels of optimization, leading to a faster convergence process. Besides, PRO10 provides some additional new features, including air cavity correction, the intermediate dose option, and jaw tracking. Investigations by Vanetti et al.^(6)^ comparing the performance of the optimization algorithms for a series of different clinical cases showed that the plan quality was generally improved with PRO10 compared to PRO8. The optimization algorithms were described in details in previous investigations.^(^ ^6^ ^,^ ^14^, ^15^, ^16^ ^)^ The current study focused on the role of the air cavity correction option and the intermediate dose option of PRO10 on the plan quality for targets with heterogeneities. The air cavity correction is an additional parameter for fine‐tuning the inhomogeneity correction by applying finer resolution to calculate the scatter component. It can influence the results only when used together with inhomogeneity correction during optimization. When the air cavity correction option is turned on, the dose in an air cavity, or other low‐density medium structure calculated by MRDC, will be smaller than that calculated without this option. With the intermediate dose option, a correction map is determined between the results of the final dose calculation and the MRDC at the end of the first optimization loop. It is possible for the users to optimize the plan, calculate the dose with the final algorithm, and then use the calculated dose as reference dose for a short second run of optimization. This option is useful when the dose‐volume histogram (DVH) calculated during arc optimization deviates from the one produced during the final dose calculation, especially when there is heterogeneity in the area to be treated.

### B. Assessment of optimization performance using virtual phantoms

**Figure 1:**
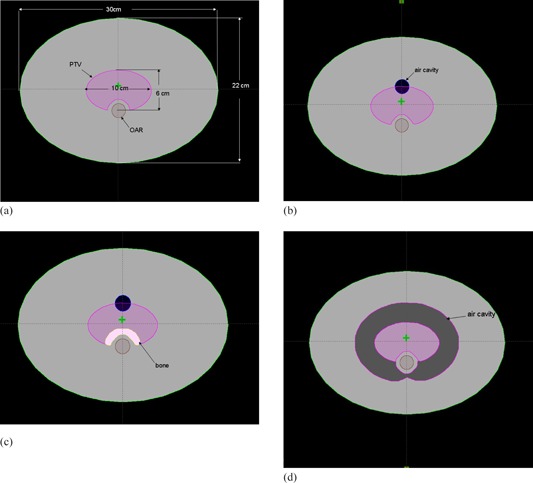
The axial view of the central slice for (a) the homogeneous phantom A, (b) phantom B with small air cavity, (b) phantom C with small air cavity and bone, and (d) phantom D with large air cavity surrounding the PTV.

Four heterogeneous virtual phantoms were created using the Eclipse planning system (Eclipse 10.0.25; Varian Medical Systems). The contours of the external body and the organs at risk (OAR) were the same on each phantom. The two‐dimensional contours of the organs at the central slice were extended in the superior‐inferior directions, with the axial views shown in Fig. 1. The length of the external body was 30 cm with an ellipsoid cross‐sectional area. Phantom A was a homogeneous phantom with water‐equivalent density, as shown in Fig. 1(a). A small air cavity with a volume of 23.3 cm^3^ and a density of 0.0012g/cm^3^ was contoured near and within the PTV of phantom B, as shown in Fig. 1(b). The same small air cavity as in phantom B and an additional bony structure with a volume of 50.5 cm^3^ and a density of 1.85 g/cm^3^ was contoured within the PTV of phantom C, as shown in Fig. 1(c). A relative large air cavity with a volume of 1680 cm^3^ surrounding the PTV was contoured in phantom D, as shown in Fig. 1(d). For all phantoms, the planning goal was to give at least 95% of the PTV a prescribed dose of 70 Gy in 35 fractions and $D_{1\%}$ (the dose encompassing 1% of the volume) of OAR less than 58 Gy. The performance of optimization was compared between PRO10 using air cavity correction option (PRO10_air) and without using air cavity correction option (PRO10_no‐air). The effect of using intermediate dose option was also compared between PRO10_no‐air and PRO10_air. The dose volume constraints, priorities, and arc geometries were initially defined using PRO10_no‐air based on AXB dose calculations to achieve a satisfactory plan quality for the phantom A geometry. The same planning parameters and optimization objectives were kept the same for all the other phantoms for both PRO10_no‐air and PRO10_air. Only one single optimization cycle was performed for each plan. No interactive action was performed during the optimization process. The comparison of plan quality between the two optimization options was, therefore, independent of operator skill. For the group of plans using intermediate dose option, a fast second optimization cycle was performed using the final calculated dose result of the first cycle as the reference dose.

### C. Assessment of optimization performance using clinical cases

#### C.1 NPC cases

Twelve patients having NPC with different staging (T1 to T4) were selected for this study. The target volumes of NPC usually included small volumes of air cavities and bony structures.

Multiple dose levels to PTVs were achieved using simultaneous integrated boost technique. Seventy (70) Gy was prescribed to ${\text{PTV}}_{70}$ that included the primary gross tumor volume and the nodal gross tumor volume encompassed positive lymph nodes. Sixty (60) Gy was prescribed to ${\text{PTV}}_{60}$ that included the high‐risk clinical target volume and the nodal clinical target volume. Fifty‐four (54) Gy was prescribed to ${\text{PTV}}_{54}$ that included the low‐risk clinical target volume. The RA plans were created using three arcs, with two complete arcs, each with 358° arc length plus one partial arc with 240° arc length. Nonzero collimator angles and zero couch angles were used for all RA plans. The optimization goal was to ensure at least 95% of the PTVs to receive the prescribed dose, and no more than 5% of the ${\text{PTV}}_{70}$ would receive 107% of the prescribed dose while minimizing the doses to OARs.

#### C.2 NSCLC cases

Ten patients having NSCLC were selected for this study because the target volume unavoidably included some lung tissues and mostly surrounded by a large volume of low‐density lung. Contouring of internal target volumes (ITV) was based on the maximum intensity projection (MIP) computed tomographic (CT) dataset of all breathing phases derived using fourdimensional CT scans with a GE LightSpeed RT 16 multislice CT simulator (GE Healthcare, Waukesha, WI). A PTV margin of 5 mm was contoured around the ITV to take into account the set‐up uncertainty, small delineating uncertainty, as well as intrafractional variations in tumor motion. The OARs, such as the normal lung, heart, spinal cord, proximal bronchial tree (PBT) and esophagus, were contoured based on the average CT dataset (AVE) of all breathing phases. A dose of 60 Gy was prescribed at the 90% isodose line and was given in 5 fractions within 14 days, with interfraction interval between 40 to 96 hours apart. The RA plans were created using three partial arcs, with about 210° to 230° arc length. The arc angles are selected to avoid the contralateral lung. The optimization goal was to ensure at least 95% of the PTV to receive the prescribed dose, and the PTV maximum dose be within the range from 110% to 140% of the prescribed dose while minimizing the doses to OARs.

The same optimization template was used in both PR010_no‐air and PR010_air for each patient in section C.1 and C.2. For each plan, only one single optimization cycle was performed without interactive action during the optimization process. The effect of the intermediate dose option in PR010 was tested for NSCLC cases only.

### D. Evaluation of plan quality

The parameters used for evaluating the plan quality among different optimization options included the target coverage in tissue components of different densities, target conformity, target dose homogeneity, and critical organ sparing. The target coverage was evaluated using the percentage volume of the PTV receiving 100% of the reference dose, $V_{100\%}$. The plan conformity was evaluated using the confirmation number, CN, which was defined as the product of $V_{T,\text{ref}}/V_{T}$ and $V_{T,\text{ref}}/V_{\text{ref}}$, where $V_{T,\text{ref}}$ represents the volume of the target receiving a dose equal to or greater than the reference dose, $V_{T}$ represents the physical volume of the target, and $V_{\text{ref}}$ represents the total tissue volume receiving a dose equal to or greater than the reference dose. ^(17)^ The reference dose used to compute the CN is the prescription dose. A higher CN indicates better conformity. Target dose homogeneity was evaluated by using the homogeneity index (HI) defined as the ratio $(D_{2\%}-D_{98\%})/D_{50\%}$,^(18)^ where $D_{x\%}$ is the dose received by at least x% of the corresponding volume. A lower HI indicates a more homogeneous target dose. For the OARs in NPC cases, the mean dose ($D_{\text{mean}}$) to the parotid glands, the $D_{1\%}$ values to brain stem, spinal cord, optic chiasm, optic nerve, and temporal lobe were reported. For OARs in NSCLC cases, the volume of lung excluding the GTV (Lung‐GTV) that received more than 20 Gy $\left(V_{20\text{Gy}}\right)$, the $D_{1\%}$ values to heart, trachea, esophagus, proximal bronchial tree (PBT), and spinal cord were reported. Parameters estimated by the mathematical formulas similar to those reported in Vanetti et al.^(6)^ were used to interpret the comparison results. For the comparison of CN values of PRO_x against PRO_y (i.e. PRO_x vs. PRO_y), the result was represented by manipulating the CN values using the formula, 1+(PRO_y−PRO_x). For HI, it was 1+(PRO_x−PRO_y). For target coverage ($V_{100\%}$), it was 1+(PRO_y−PRO_x)/100. For $D_{1\%}$ and $D_{\text{mean}}$ to OAR, it was 1+(PRO_x−PRO_y)/100. By using the above formulas, any parameter with value greater than 1 indicates a better result by using PRO_y. In addition, the Wilcoxon matched pair signed‐rank test was used to compare the results between the above two couples for clinical cases. The threshold for statistical significance was p<0.05. All statistical tests were two‐sided, and all analyses were performed using the Statistical Package for Social Sciences, version 11.0 (SPSS, Chicago, IL).

## III. RESULTS

### A. Comparison Results using Virtual Phantoms

**Figure 2:**
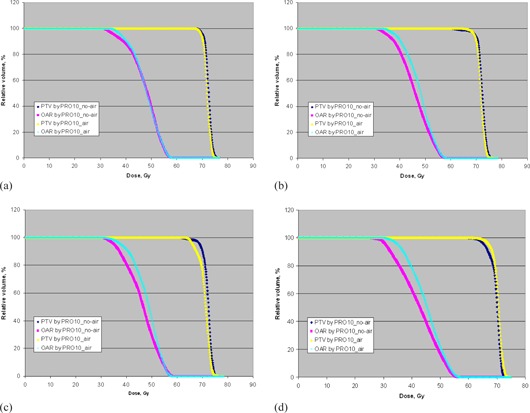
The dose‐volume histograms of the PTV and OAR for phantoms A (a), B (b), C (c), and D (d) optimized by PR010 no‐air and PRO10 air based on AXB dose calculations.

Table 1 summarizes the results of target conformity, dose homogeneity, target coverage of different density components, and doses to OAR for phantoms A to D, based on the AXB dose calculation. Figures 2(a) to 2(d) also show the corresponding dose‐volume histograms (DVHs) of the PTV and OAR for phantoms A to D. For the homogeneous phantom A, the planning results of PRO10_air and PRO10_no‐air were very close to each other, with PRO10_air produced 1.8% lower coverage to the PTV. For the heterogeneous phantoms B to D, PRO10_air produced about 4% to 5% higher mean doses to the OAR than PRO10_no‐air. For phantom B, in which only a small air cavity was present, PRO10_air produced more than 10% higher coverage to the PTV in air than PRO10_no‐air. However, it produced 3% lower coverage to the PTV in tissue and 2% lower coverage to the overall PTV, resulting in an inferior overall target conformity. For phantom C in which both small air and bone were present within the PTV, PRO10_air produced about 13% lower target coverage and inferior conformity than

**Table 1:** Dosimetric evaluation of virtual phantoms with heterogeneous media based on AXB dose calculations

	*PROW no‐air*	*PRO10_air*	*PRO10 air vs. PRD10_no‐air*
*Phantom A: Homogeneous target*			
CN(PTV)	0.93	0.91	1.02
HI(PTV)	0.08	0.08	1.00
$\text{Covg}(\text{PTV}),\;V_{100\%}$,	95.80	94.00	1.02
$\text{OAR},\;D_{1\%},\;\text{Gy}$	56.90	57.10	1.00
*Phantom B: Target with small air cavity*			
CN(PTV)	0.91	0.90	1.01
HI(PTV)	0.11	0.09	0.98
$\text{Covg}(\text{PTV}),\;V_{100\%}$,%	93.60	91.30	1.02
$\text{Covg}(\text{PTV}\_\text{tissue}),\;V_{100\%},\;\%$	95.70	92.80	1.03
$\text{Covg}(\text{PTV}\_\text{air}),\;V_{100\%}$,%	29.90	41.60	0.88
$\text{OAR},\;D_{1\%},\;\text{Gy}$	56.80	57.10	1.00
*Phantom C: Target with small air cavity and bone*			
CN(PTV)	0.88	0.75	1.13
HI(PTV)	0.11	0.13	1.02
$\text{Covg}(\text{PTV}),\;V_{100\%},\;\%$	90.10	76.60	1.14
$\text{Covg}(\text{PTV}\_\text{tissue}),\;V_{100\%},\;\%$	92.20	77.70	1.15
$\text{Covg}(\text{PTV}\_\text{bone}),\;V_{100\%},\;\%$	61.40	3.90	1.58
$\text{Covg}(\text{PTV}\_\text{air}),\;V_{100\%},\;\%$	21.30	38.10	0.83
$\text{OAR},\;D_{1\%},\;\text{Gy}$	57.20	56.90	1.00
*Phantom D: Target surrounded by large air cavity*			
CN(PTV)	0.53	0.67	0.86
HI(PTV)	0.12	0.10	0.98
$\text{Covg}(\text{PTV}),\;V_{100\%},\;\%$	53.70	67.50	0.86
$\text{OAR},\;D_{1\%},\;\text{Gy}$	55.00	55.60	1.01

PRO10_no‐air. Although using air cavity correction in PRO10 increased the target coverage in air, the coverage in bone was significantly decreased by about 58%, leading to a decrease in the overall target coverage and conformity. For phantom D in which the PTV was surrounded by a large volume of air, PRO10_air produced 14% higher target coverage and better conformity than PRO10 no‐air.

Table 2 summarizes the plan comparison results between PRO10_air and PRO10_no‐air for phantoms A to D based on the AAA dose calculation. It was observed that the target coverage and OAR doses were generally increased for all phantom plans after recalculations by AAA. The amounts of increase in dose were larger in the target components of air and bone than those of tissue. When looking at the values of the parameters for PRO10_air vs. PRO10_no‐air, the differences between the two options were in general minimized, but the use of the air cavity correction option induced a similar trend to that observed for the AXB calculations. It improved the target coverage and conformity for phantom D. For phantoms C, it increased the target coverage in air and decreased the target coverage in bone, with the amount of changes less pronounced compared to those observed based on the AXB calculation.

**Table 2:** Dosimetric evaluation of virtual phantoms with heterogeneous media based on AAA dose calculations

	*PRO10 no‐air*	*PRO10_air*	*PRO10 air vs. PRO10 no‐air*
*Phantom A: Homogeneous target*			
CN(PTV)	0.94	0.93	1.01
HI(PTV)	0.08	0.08	1.00
$\text{Covg}(\text{PTV}),\;V_{100\%},\;\%$	97.88	96.83	1.01
$\text{OAR},\;D_{1\%},\;\text{Gy}$	57.82	57.92	1.00
*Phantom B: Target with small air cavity*			
CN(PTV)	0.95	0.94	1.01
HI(PTV)	0.08	0.08	1.00
$\text{Covg}(\text{PTV}),\;V_{100\%},\;\%$	97.24	96.78	1.00
$\text{Covg}(\text{PTV}\_\text{tissue}),\;V_{100\%},\;\%$	97.68	96.68	1.01
$\text{Covg}(\text{PTV}\_\text{air}),\;V_{100\%},\;\%$	86.77	100.00	0.86
$\text{OAR},\;D_{1\%},\;\text{Gy}$	57.60	57.85	1.00
*Phantom C: Target with small air cavity and bone*			
CN(PTV)	0.95	0.90	1.05
HI(PTV)	0.08	0.09	1.02
$\text{Covg}(\text{PTV}),\;V_{100\%},\;\%$	97.82	92.72	1.05
$\text{Covg}(\text{PTV}\_\text{tissue}),\;V_{100\%},\;\%$	98.12	92.5	1.06
$\text{Covg}(\text{PTV}\_\text{bone}),\;V_{100\%},\;\%$	98.45	66.30	1.32
$\text{Covg}(\text{PTV}\_\text{air}),\;V_{100\%},\;\%$	91.32	100.0	0.91
$\text{OAR},\;D_{1\%},\;\text{Gy}$	58.03	57.64	1.01
*Phantom D: Target surrounded by large air cavity*			
CN(PTV)	0.77	0.85	0.92
HI(PTV)	0.12	0.11	0.98
$\text{Covg}(\text{PTV}\_\text{air}),\;V_{100\%},\;\%$	77.41	85.54	0.92
$\text{OAR},\;D_{1\%},\;\text{Gy}$	56.06	56.59	1.01

**Figure 3:**
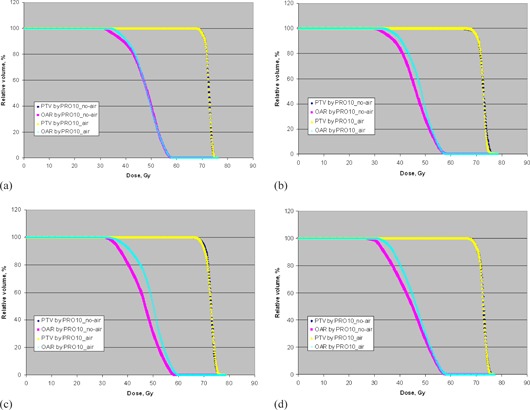
The dose‐volume histograms of the PTV and OAR for phantoms A (a), B (b), C (c), and D (d) optimized by PRO10_no‐air and PRO10_air after using the intermediate dose option in the second run of optimization based on AXB dose calculations.

Figures 3(a) to 3(d) show the DVH comparison of the PTV and OAR between PRO10_air and PRO10_no‐air for phantoms A to D after using the intermediate dose option in the second run of optimization based on the AXB dose calculations. It is observed that the use of the intermediate dose option significantly minimize the difference in target coverage between PRO10_air and PRO10_no‐air, but the difference in OAR sparing remained to be similar to that before using the intermediate dose option.

### B. Comparison Results for NPC Cases

**Figure 4:**
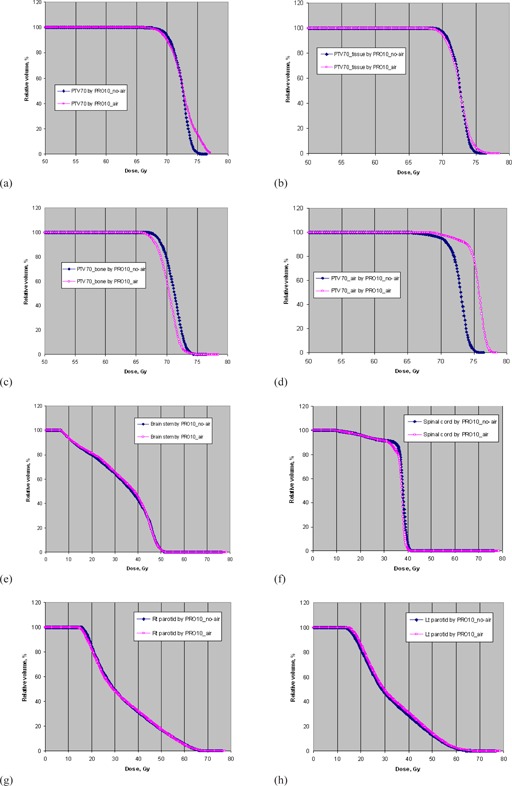
The dose‐volume histograms of: (a) the originally contoured ${\text{PTV}}_{70}$, (b) ${\text{PTV}}_{70}$ in tissue, (c) ${\text{PTV}}_{70}$ in bone, (c) ${\text{PTV}}_{70}$ in air, (e) brain stem, (f) spinal cord, (g) right parotid, and (h) left parotid optimized PRO10_no‐air and PRO 10_air for a typical NPC patient based on AXB dose calculations.

Table 3 summarizes the comparison results of target conformity, dose homogeneity, coverage, and doses to OARs averaged over the 12 NPC cases based on AXB dose calculations. Figures 4(a) to 4(h) show the corresponding DVHs of the originally contoured ${\text{PTV}}_{70},{\text{PTV}}_{70}$ in different density components and some OARs for a typical patient. It was observed that for ${\text{PTV}}_{70}$, the conformity decreased from 0.81 to 0.77, the overall target coverage was decreased by 3%, and the HI value increased from 0.08 to 0.1 when air cavity correction option was turned on for optimization. PRO10_air produced 1% lower coverage to the ${\text{PTV}}_{70}$ in tissue and 17% lower coverage to the ${\text{PTV}}_{70}$ in bone than PRO10_no‐air. It can be seen in Fig. 4(a) that the use of PRO10_air produced more hot areas in ${\text{PTV}}_{70}$. Although PRO10_air produced about 4% to 5% higher target coverage in air than PRO10_no‐air, this was not clinically beneficial. It produced inferior coverage to the ${\text{PTV}}_{70}$ in bone than PRO10_no‐air, as shown in Fig. 4(c). For ${\text{PTV}}_{60}$, the differences between the two options were minimized because of the smaller proportion of heterogeneous media. The sparing of OARs produced by the two optimization options was similar to each other. Based on the AXB dose calculations, it was obvious that the use of air cavity correction option produced inferior plan quality.

**Table 3:** Plan comparison between PRO10no‐air and PRO10 air for NPC cases (average over 12 patients) based on AXB dose calculations

	*PRO10 no‐air*	*PRQ10_air*	*PRD10 air vs. PRO10 no air, p‐values* ^a^
*PTV*			
$\text{CN}\;({\text{PTV}}_{70})$	0.81±0.04	0.77±0.04	1.04±0.02,+
$\text{HI}\;({\text{PTV}}_{70})$	0.08±0.02	0.10±0.02	1.02±0.01,+
$\text{Covg}({\text{PTV}}_{70}),\;V_{100\%},\;\%$	93.82±3.23	90.89±2.64	1.03±0.02,+
$\text{Covg}({\text{PTV}}_{70}\_\text{tissue}),\;V_{100\%},\;\%$	97.17±2.74	95.91±2.89	1.01±0.01,+
$\text{Covg}({\text{PTV}}_{70}\_\text{bone}),\;V_{100\%},\;\%$	85.10±5.31	67.59±6.76	1.18±0.04,+
$\text{Covg}({\text{PTV}}_{70}\_\text{air}),\;V_{100\%},\;\%$	93.85±5.17	99.25±1.23	0.95±0.04,+
$\text{CN}\;({\text{PTV}}_{60})$	0.85±0.02	0.84±0.02	1.00±0.00,~
$\text{HI}\;({\text{PTV}}_{60})$	0.21±0.01	0.22±0.01	1.01±0.01,+
$\text{Covg}({\text{PTV}}_{60}),\;V_{100\%},\;\%$	98.14±0.50	98.00±0.81	1.00±0.01,~
$\text{Covg}({\text{PTV}}_{60}\_\text{tissue}),\;V_{100\%},\;\%$	98.96±0.46	98.91±0.50	1.00±0.00,~
$\text{Covg}({\text{PTV}}_{60}\_\text{bone}),\;V_{100\%},\;\%$	96.58±0.99	93.76±1.62	1.03±0.01,+
$\text{Covg}({\text{PTV}}_{60}\_\text{air}),\;V_{100\%},\;\%$	94.93±2.24	98.77±0.61	0.96±0.02,+
*OAR*			
Brain stem, $D_{1\%},\;\text{Gy}$	49.67±1.57	49.41±1.63	1.00±0.01,~
Spinal cord, $D_{1\%},\;\text{Gy}$	39.65±0.97	39.01±1.09	0.99±0.00,+
Optic chiasma, $D_{1\%},\;\text{Gy}$	23.65±18.21	23.93±19.31	1.00±0.00,~
Optic nerve, $D_{1\%},\;\text{Gy}$	23.2±18.09	23.66±18.48	1.00±0.01,+
Temporal lobe, $D_{1\%},\;\text{Gy}$	63.16±3.49	62.18±3.46	0.99±0.01,+
Parotid gland, $D_{\text{mean}},\;\text{Gy}$	31.83±2.66	32.15±2.75	1.00±0.00,+

a “+” represents a p‐value ≤0.05, while “~” represents a p‐value > 0.05.

Table 4 summarizes the comparison results of target conformity, dose homogeneity, coverage, and doses to OARs averaged over the 12 NPC cases when all the plans were recalculated using AAA. The target coverage and OAR doses were in general increased and the target conformation number was decreased after the recalculation by AAA, as compared to those calculated by AXB. Although it was observed that for ${\text{PTV}}_{70}$, using PRO10_air increased the conformity slightly from 0.73 to 0.75, the HI values and the overall target coverage remained almost the same as those using PRO10_no‐air. The use of PRO10_air decreased the coverage to ${\text{PTV}}_{70}$ in bone by about 2.5% compared to PRO10_no‐air, a much smaller extent when compared to the results of the AXB dose calculations. On the other hand, the use of PRO10_air increased the coverage to ${\text{PTV}}_{70}$ in air by about 10%, which was more pronounced when compared to the results of the AXB dose calculations. The sparing of OARs produced by the two optimization options was similar to each other. On the whole, based on the AAA dose calculations, the use of the air cavity correction option showed little improvement on the plan quality to NPC cases.

**Table 4:** Plan comparison among PRO10_no‐air and PRO10_air for NPC cases (average over 12 patients) based on AAA dose calculations

	*PRO10 no‐air*	*PRO10_air*	*PRO10 air vs. PRO10 no air, p‐values* ^a^
*PTV*			
$\text{CN}\;({\text{PTV}}_{70})$	0.73±0.07	0.75±0.06	0.98±0.02,+
$\text{HI}\;({\text{PTV}}_{70})$	0.08±0.02	0.08±0.02	1.00±0.01,~
$\text{Covg}({\text{PTV}}_{70}),\;V_{100\%},\;\%$	96.79±2.82	97.45±2.09	0.99±0.02,~
$\text{Covg}({\text{PTV}}_{70}\_\text{tissue}),\;V_{100\%},\;\%$	98.68±1.74	98.70±1.66	1.00±0.00,~
$\text{Covg}({\text{PTV}}_{70}\_\text{bone}),\;V_{100\%},\;\%$	98.77±1.29	96.21±2.32	1.03±0.02,+
$\text{Covg}({\text{PTV}}_{70}\_\text{air}),\;V_{100\%},\;\%$	87.67±8.95	97.50±2.03	0.90±0.08,+
$\text{CN}\;({\text{PTV}}_{60})$	0.82±0.02	0.82±0.02	1.00±0.01,~
$\text{HI}\;({\text{PTV}}_{60})$	0.21±0.01	0.21±0.01	1.01±0.00,~
$\text{Covg}({\text{PTV}}_{60}),\;V_{100\%}$ %	98.77±0.36	99.55±0.4	1.00±0.00,~
$\text{Covg}({\text{PTV}}_{60}\_\text{tissue}),\;V_{100\%},\;\%$	99.20±0.32	99.19±0.32	1.00±0.00,~
$\text{Covg}({\text{PTV}}_{60}\_\text{bone}),\;V_{100\%},\;\%$	99.25±0.56	98.12±1.02	1.01±0.01,+
$\text{Covg}({\text{PTV}}_{60}\_\text{air}),\;V_{100\%},\;\%$	95.51±1.81	99.02±0.62	0.96±0.01,+
*OAR*			
Brain stem, $D_{1\%},\;\text{Gy}$	50.56±1.62	50.25±1.72	1.00±0.01,~
Spinal cord, $D_{1\%},\;\text{Gy}$	40.52±1.13	39.98±1.20	0.99±0.00,+
Optic chiasma, $D_{1\%},\;\text{Gy}$	24.22±18.19	24.45±18.26	1.00±0.00,~
Optic nerve, $D_{1\%},\;\text{Gy}$	23.83±18.07	24.29±18.43	1.00±0.01,+
Temporal lobe, $D_{1\%},\;\text{Gy}$	63.20±3.64	62.31±3.60	0.99±0.01,+
Parotid gland, $D_{\text{mean}},\;\text{Gy}$	32.94±2.66	33.27±2.77	1.00±0.00,+

a “+” represents a p‐value ≤0.05, while “~” represents a p‐value > 0.05.

### C. Comparison results for NSCLC cases

**Figure 5:**
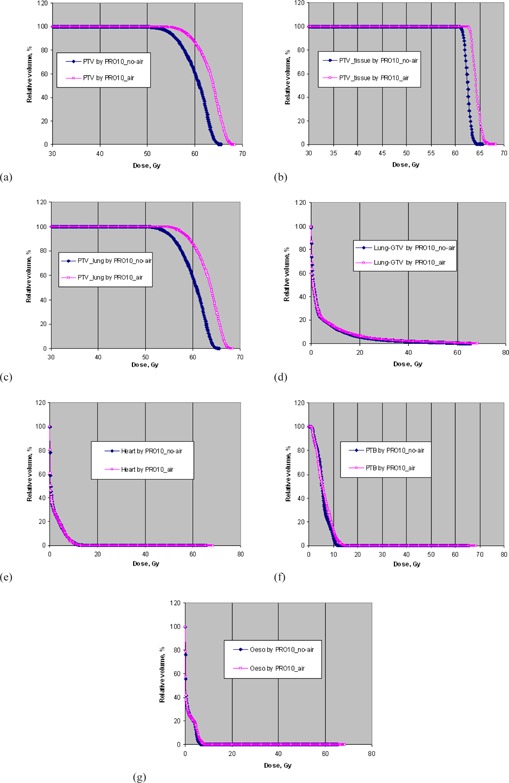
The dose‐volume histograms of: (a) the originally contoured PTV, (b) PTV in tissue, (c) PTV in lung, (d) lung‐GTV, (e) heart, (f) PTB, and (g) esophagus optimized PRO10_no‐air and PRO10_air for a typical NSCLC patient based on AXB dose calculations.

Table 5 summarizes the comparison results of target conformity, dose homogeneity, coverage and doses to OARs averaged over the ten NSCLC cases based on the AXB calculations. Figures 5(a) to 5(g) show the corresponding DVHs of the originally contoured PTV, PTV in tissue, PTV in lung, and some OARs for a typical patient. PRO10_air produced better conformity, target dose heterogeneity, and target coverage, especially PTV in lung, compared to PRO10_no‐air. The use of the air cavity correction option increased the CN from 0.72 to 0.8, the overall coverage to PTV by 15% and the coverage to PTV in lung by 20%. At the same time, using PRO10_air unavoidably delivered about 5% to 6% more doses to the heart and lung‐GTV. Table 6 shows the comparison results of plan quality between PRO10_air and PRO10_no‐air after recalculations by AAA. The effect of using the air cavity correction option was very close to those using the AXB dose calculations. Table 7 shows the comparison results of plan quality between PRO10_air and PRO10_no‐air after using the intermediate dose option in the second run of optimization. The results showed that there was negligible difference between the plan quality with and without using the air cavity correction option for PRO10.

**Table 5:** Plan comparison between PRO10_no‐air and PRO10_air for NSCLC cases (average over 10 patients) based on AXB dose calculations

	*PRO10 no‐air*	*PRO10_air*	*PRO10 air vs. PRO10 no air, p‐values* ^a^
*PTV*			
CN(PTV)	0.73±0.09	0.82±0.05	0.91±0.06,+
HI(PTV)	0.20±0.04	0.17±0.04	0.98±0.01,+
$\text{Covg}(\text{PTV}),\;V_{100\%},\;\%$	78.00±10.02	92.32±4.15	0.86±0.06,+
$\text{Covg}(\text{PTV}\_\text{tissue}),\;V_{100\%},\;\%$	98.87±1.45	99.65±0.38	0.99±0.01,+
$\text{Covg}(\text{PTV}\_\text{lung}),\;V_{100\%},\;\%$	68.92±13.16	89.54±6.10	0.79±0.08,+
*OAR*			
Spinal cord, $D_{1\%},\;\text{Gy}$	12.49±5.06	13.24±4.76	1.01±0.01,~
Heart, $D_{1\%},\;\text{Gy}$	16.65±9.58	17.56±9.90	1.01±0.01,~
$\text{Lung}‐\text{GTV},\;V_{20\text{Gy}},\;\%$	7.83±2.59	8.32±2.69	1.00±0.00,+
$\text{PBT},\;D_{1\%},\;\text{Gy},$	6.96±6.02	6.99±5.72	1.00±0.02,~
Trachea, $D_{1\%},\;\text{Gy}$	3.98±6.90	4.10±7.18	1.00±0.00,~
Esophagus, $D_{1\%},\;\text{Gy}$	14.29±5.21	15.07±5.96	1.01±0.02,~

a “+” represents a p‐value ≤0.05, while “~” represents a p‐value > 0.05.

**Table 6:** Plan comparison between PRO10_no‐air and PRO10_air for NSCLC cases (average over 10 patients) based on AAA dose calculations

	*PRO10 no‐air*	*PRO10_air*	*PRO10_air vs PRO10 no air, p‐values* ^a^
*PTV*			
CN(PTV)	0.72±0.07	0.83±0.04	0.90±0.07,+
HI(PTV)	0.17±0.02	0.16±0.03	0.98±0.01,+
Covg(PTV), %	77.62±8.13	93.26±2.78	0.84±0.06,+
$\text{Covg}(\text{PTV}\_\text{tissue}),\;V_{100\%},\;\%$	99.02±1.41	99.58±0.64	1.00±0.01,+
Covg(PTV_lung), $V_{100\%}$, %	70.53±12.19	91.86±3.62	0.79±0.09,+
*OAR*			
Spinal cord, $D_{1\%},\;\text{Gy}$	13.50±4.90	14.30±4.50	1.01±0.01,~
Heart, $D_{1\%},\;\text{Gy}$	16.90±9.76	17.90±10.08	1.01±0.01,~
Lung‐GTV, $V_{20\text{Gy}}$, %
PBT, $D_{1\%}$, Gy,	7.76±2.59	8.26±2.69	1.01±0.00,+
	7.25±6.17	7.22±5.97	1.00±0.02,~
Trachea, $D_{1\%},\;\text{Gy}$	3.99±6.91	4.13±7.19	1.00±0.00,~
Esophagus, $D_{1\%},\;\text{Gy}$	14.50±5.33	16.37±6.03	1.01±0.02,~

a “+” represents a p‐value ≤0.05, while “~” represents a p‐value > 0.05.

**Table 7:** Plan comparison between PRO10_no‐air and PRO10_air for NSCLC cases after using intermediate dose option based on AXB dose calculations

	*PRO10 no‐air*	*PRO10_air*	*PRO10_air vs PRO10 no air, p‐values* ^a^
*PTV*			
CN(PTV)	0.82±0.11	0.81±0.11	1.01±0.02,~
HI(PTV)	0.11±0.01	0.11±0.02	0.99±0.01,~
$\text{Covg}(\text{PTV}),\;V_{100\%},\;\%$	99.60±0.55	99.80±0.25	1.00±0.01,~
$\text{Covg}(\text{PTV}\_\text{tissue}),\;V_{100\%},\;\%$	99.94±0.11	99.96±0.06	1.00±0.00,~
$\text{Covg}(\text{PTV}\_\text{lung}),\;V_{100\%},\;\%$	99.39±0.89	99.69±0.31	1.00±0.01,~
*OAR*			
Spinal cord, $D_{1\%},\;\text{Gy}$	14.39±4.30	14.87±4.34	1.00±1.00,~
Heart, $D_{1\%},\;\text{Gy}$	18.39±10.47	18.52±10.38	1.00±0.01,~
Lung‐GTV, $V_{20\text{Gy}}$ % PBT, $D_{1\%},\;\text{Gy}$	8.90±2.75	9.01±2.81	1.00±0.00,~
	8.14±6.89	8.32±7.05	1.00±0.02,~
Trachea, $D_{1\%},\;\text{Gy}$	4.58±7.83	4.51±7.81	1.00±0.00,~
Esophagus, $D_{1\%},\;\text{Gy}$	15.84±5.38	16.21±5.85	1.00±0.02,~

a “+” represents a p‐value ≤0.05, while “~” represents a p‐value > 0.05.

## IV. DISCUSSION

From the results of phantom D and also those of the NSCLC cases, it was observed that when the target was close to or surrounded by a large volume of low‐density medium such as air or lung, the target coverage would be significantly improved with the use of air cavity correction option of PRO10 compared to that without using it. This was because with the use of the air cavity correction option, a lower dose would be calculated by MRDC in the low‐density medium. In order to meet optimization criteria, this would then force the optimization algorithm to deliver more doses to and near the low‐density media. Based on the AXB dose calculation, the target coverage of phantoms A and D achieved by PRO10_air was 94% and 67.5%, respectively. Since the same optimization objectives were used for both phantoms, the significantly lower coverage achieved in phantom D indicated that even with the use of air cavity correction, the dose changes due to the presence of low‐density media was not fully modeled as in Acuros XB during optimization. There are two ways to solve this problem. The first method was to set a higher dose to PTV in the optimization objective, but the value to achieve a satisfactory plan had to be found by trial and error. For example, a dose 5% to 10% higher than the prescribed dose was set by the authors to PTV for the NSCLC cases in this study, such that the target coverage achieved by PRO10_air was quite acceptable. The same optimization objectives used in PRO10_air for PRO10_no‐air resulted in significantly lower target coverage. That means the dose set in the optimization objective for PTV should be increased if PRO10_no‐air is used. The second method was to perform a second run of optimization using the intermediate dose option. Our results showed that this was a very effective way to improve the conformity and target coverage. The current study also proved that if the user determined to use the intermediate dose option, whether or not the air cavity correction option is used would have little influence on the planning result.

Based on the AXB dose calculations, the planning results of phantom C and those of the NPC cases showed that for cases with small volumes of both air and bone, PRO10_no‐air produced superior plan quality compared to PRO10_air. The use of air cavity correction option in PRO10 was not beneficial. In general, when compared to PRO10_no‐air, the use of PRO10_air increased the target coverage in air, slightly decreased the target coverage in tissue, and significantly decreased the target coverage in bone. It should be noted that although air is unavoidably contoured in many head and neck cases, the increase of dose to air is in fact clinically irrelevant. On the other hand, the decrease in dose to tissue and bone due to the use of air cavity correction option is undesirable. The significant reduction of doses to bone due to the use of air cavity correction was not clear. This might be because when this option is turned on, the dose in bony structure calculated by MRDC will be higher than that obtained without this option. The optimization process will then result in lower doses to bone after the final dose calculation by Acuros XB.

For NPC cases, the use of intermediate dose option was not included in the current study. This was because preliminarily tests performed by the authors showed that this option would not produce as much improvement in NPC cases as in lung cases. When looking at the target conformity, dose homogeneity, the target coverage, and organ sparing in Table 4, the plans obtained with PRO10_no‐air met most optimization criteria and would have been considered to be very close to clinically acceptable quality. It should be borne in mind that these plans were generated using only one single optimization without any interactive action. A few more optimization cycles would further improve the plan quality to a satisfactory one. The comparison between the optimization options did not depend on the skill of the planner as the same optimization template was used for both options. However, the ease of achievement of satisfactory plans depends on the experience of the planner. The optimization templates of all the plans were generated by an experienced physicist.

The plan comparison results were initially generated and optimized based on the AXB dose calculation. Although AAA is less accurate compared to AXB in terms of dose calculation in a heterogeneous medium, it is still a very commonly used dose calculation algorithm in the clinical environment. The authors therefore recalculated all plans using AAA to investigate whether there was any change under the influence of the air cavity correction option due to the conversion of dose calculation method. It was found that for the NSCLC cases, almost the same observation was found as for the AXB dose calculations. For NPC cases, some differences were found. Based on the AAA dose calculation, quite equivalent quality was found between plans with and without using the air cavity correction option, while based on the AXB dose calculation, a more significant decrease in the coverage to the PTV, especially in the bony component, was observed when using the air cavity correction option in PRO. This was due to fact that AXB would calculate lower doses in bony structures than AAA. AAA computed and reported the absorbed dose in bone as if it were deposited in water, while AXB calculated dose considering the elemental composition. The bone was different from water in both density and atomic number. A stopping power ratio of water to cortical bone is about 1.1. Our previous investigation observed that there could be about 4% reduction of minimum doses in the bony content of the PTVs when RapidArc plans originally generated and calculated by AAA were recalculated by AXB for NPC cases.^(19)^ Nevertheless, the use of the air cavity correction option did not produce much improvement in the plan quality for NPC cases even when the dose calculation was converted from AXB to AAA. In the current study, recalculation by AAA was not performed for investigating the effect of using the intermediate dose option, but the authors believed that the results would be similar to those using AXB dose calculations.

## V. CONCLUSIONS

When using the same optimization template, the use of the air cavity correction option in PRO10 improved the target coverage for cases that involved a large volume of low‐density media in or adjacent to the target volume, such as lung cancer. However, when the intermediate dose option was used in the second run of optimization, there would be little difference between PRO10_no‐air and PRO10_air. For NPC cases or cases for which small volumes of both low‐ and high‐density media were present in the target volume, using PRO10_air produced slightly inferior target coverage and conformity than PRO10_no‐air based on the AXB dose calculation, and comparable plan quality as PRO10_no‐air based on the AAA dose calculation.
